# Sexual, reproductive and mental health among young men (10–24) in low-and-middle income countries: a scoping review

**DOI:** 10.3389/frph.2023.1119407

**Published:** 2023-12-04

**Authors:** Siphiwe Mhlongo, Amanda J. Mason-Jones, Keith Ford

**Affiliations:** Department of Health Sciences, University of York, York, England

**Keywords:** sexual and reproductive health, men’s health, mental health, adolescent, low and-middle-income countries, scoping review

## Abstract

**Background:**

The relationship between SRH and mental health among men is not well documented, especially in the 10–24 age group. This scoping review aimed to investigate what is known about the association between SRH and mental health among young men (10–24) in LMICs.

**Methods:**

Embase, APA PsycInfo, MEDLINE(R) ALL, ASSIA and the Cochrane Library of Database of Systematic Reviews were searched from the year of establishment up to August 2022. The review was reported using the PRISMA-ScR checklist.

**Results:**

A total of (*n = *2636) studies were identified from the five databases. After the completion of screening, only ten studies (*n = *8 cross-sectional, *n = *1 mixed methods and *n = *1 qualitative) met the eligibility criteria and were included in the review. The findings suggest that there is a reciprocal relationship between mental health and SRH. Sperm concentration and total sperm count were found to be lower in depressed men. Poor mental health was associated with early sexual debut, higher rates of sexual activity and an increased number of sexual partners. Poor mental health was also found among men who had sex with men (MSM). In addition, we found a relationship between sexual abuse, sexual coercion and poor mental health.

**Conclusion:**

The findings of this unique study indicate that poor mental health is associated with poor SRH outcomes and vice versa among young men (10–24) living in LMICs. However, further research will be needed to establish the temporal relationship between SRH and mental health outcomes.

## 1. Introduction

Sexual and reproductive health (SRH) needs of adolescent boys and young men (aged 10–24) often remain unmet due to issues such as limited use of SRH services, reluctance to seek help as well as poor access to services (1). Sexual and reproductive health rights (SRHR) *for all* are enshrined in 2030 Sustainable Development Goals agenda in SDGs (3.7, 4.7 and 5.6) (2). However, the majority of SRH research has focused upon health outcomes for girls and women (3) and males have not always been prioritised (4).

The limited research on the sexual and reproductive health of young men (3) largely focusses on HIV and sexually transmitted diseases. Globally, 16.8 million men are known to be living with HIV (5) and the age-standardized incidence rates of syphilis, chlamydia, gonorrhoea, and trichomoniasis are higher in males compared to females (6).

Depression and anxiety were reported to be among the top ten leading causes of the global disease burden in the 10–24 year age group (7). Although anxiety and depression rates are reported to be lower in males (13%) compared to females (20%), suicide rates in males are much higher (8). The age-standardized suicide rate for males in 2019 was 12.6 per 100,000 when compared to 5.4 per 100,000 for females (9). Approximately 77% of these deaths occurred in low-and-middle-income countries (LMICs) (9). The actual burden of mental ill health and the suicide rates in LMICs may differ due to the scarcity of empirical evidence and misclassification of deaths (9, 10). Seeking help for mental health conditions is often stigmatized in men and adolescent males (11) and may impact reporting. In addition, the majority of research on adolescents originates from high-income countries (12). Considering that around half of the population of the world is aged younger than 25 and that 90% of 10–24 year olds live in LMICs (13), this is a significant public health problem.

Although a relationship has been shown between poor mental health and sexual behaviour, such as men self-medicating using alcohol or other substances during emotional distress leading to risky sexual behaviours (5, 8, 14, 15), to the best of our knowledge no previous review has explored the association between sexual and reproductive health and mental health among young men in LMICs. Furthermore, evidence indicates that adolescents growing up in regions with higher levels of income inequality have a wide spectrum of poor health outcomes (16) which suggests that adolescent males growing up in LMICs with high levels of income inequality are at risk of experiencing poor sexual and reproductive health and mental health outcomes. Therefore, engaging males should be an important component in all programmes as it has the potential to improve the SRH and mental health of all young people (17).

Thus, considering that there is a paucity of literature on the SRH of young men and that there is potentially underreporting of mental health issues in LMICs, the interaction between these two key issues is unclear, particularly in the 10–24-year age group. To understand the potential association between SRH and mental health for young men, a scoping review (18) was conducted.

## 2. Methods

A preliminary search via Ovid on AMED, Embase, Ovid MEDLINE, APA PsycInfo and Google Scholar with the search terms “*scoping review*”, “*systematic review*”, “*sexual health*”, “*reproductive health*” and “*mental health*” was conducted to search for existing scoping or systematic reviews on the topic. There were no reviews examining the association between SRH and mental health other than one focused on women (19).

The Arksey and O’Malley framework for scoping was employed (18). The framework has five stages: (i) identifying the research question; (ii) identifying relevant studies; (iii) study selection; (iv) charting the data; and (v) collating, summarizing and reporting the results. To further strengthen the methodological rigour, the review was reported using the PRISMA-ScR checklist (20) (Supplementary Table 1). The research question was developed using the (21) framework which specified the concept, the population, and important outcomes of interest in this review. In addition, eligibility criteria were developed around the research question.

Case studies, commentaries, letters, conference abstracts and studies that were not in English were excluded. Studies were also excluded if the female population was more than 50% and if there was no association reported between SRH and mental health so that the focus of the review was maintained. To ensure that the search strategy captured a wider geographical landscape, EPOC LMIC filters were utilised to search for articles (22). However, considering that the EPOC LMIC filter list has not been recently updated, all high-income countries as per World Bank 2023 fiscal year classification (23) were removed from the list before running the search strategy. A modified two-step search strategy was then used to search for relevant articles (24). Initially, a circumscribed search was conducted on Embase and CINAHL Complete and terms contained in the titles and abstracts from the search results were analysed and used in the second step of the search strategy.

Using the initial search terms, a comprehensive search was performed on Embase, APA PsycInfo, MEDLINE(R) ALL, ASSIA and the Cochrane Library Database of Systematic Reviews up to 8 August 2022. The search results were imported into Covidence where the titles and abstracts were screened by one reviewer with a 10% random selection independently screened by two further reviewers. The following search terms were used in different combinations and adapted for each database.

• (“Reproductive health”) (“Sexual health”) OR (sexual reproductive health) OR (reproductive NEAR/2 service*) OR (reproductive health care)
• (“Mental health”) OR (mental health) OR (psychological health) OR (psychological well-being) OR (psychiatric health) OR (mental disorders) OR (mental disease)
• Juvenile OR (“Adolescents”) OR adolesce* OR teen* (“Young adults”) OR (young adulthood) OR (young pe*)
• Men OR boy* OR male* OR (male pe*)
• (LMIC) OR (low NEAR/3 countr*) OR (*insert modified EPOC LMIC FILTER LIST*)
• (“Reproductive health”) (“Sexual health”) OR (sexual reproductive health) OR (reproductive NEAR/2 service*) OR (reproductive health care) AND (“Mental health”) OR (mental health) OR (psychological health) OR (psychological well-being) OR (psychiatric health) OR (mental disorders) OR (mental disease) AND juvenile OR (“Adolescents”) OR adolesce* OR teen* (“Young adults”) OR (young adulthood) OR (young pe*) AND men OR boy* OR male* OR (male pe*) AND (LMIC) OR (low NEAR/3 countr*) OR (*insert modified EPOC LMIC filter list*)

Key information was charted in a Microsoft Word document which included the following headings: author(s), year of publication, type of publication, country/region, aims/purpose, population, outcomes, methods of measurement and key findings that related to the scoping review question. Analysis of the results utilised a three step process: (i) analysing the data; (ii) reporting the results; and (iii) applying meaning to the results (25). The findings were presented with respect to the research question and the objectives of the scoping review using graphs, figures, and tables where applicable.

## 3. Results

### 3.1. Study selection

**Figure 1:**
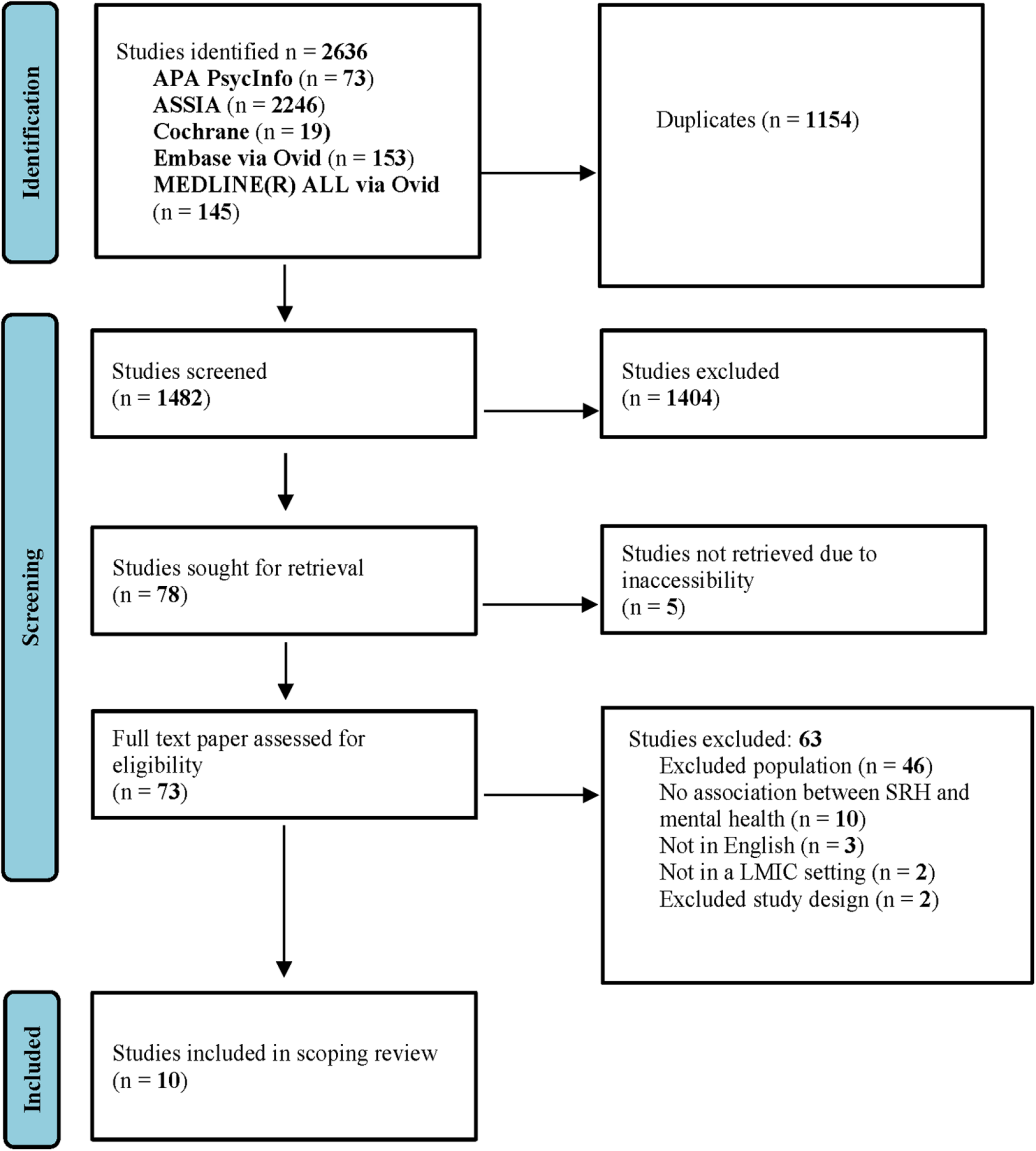
Prisma flowchart showing the identification of studies.

A total of *(n = *2,632*)* studies were identified from five databases. Altogether, *(n = *1,154*)* studies were removed as duplicates whilst *(n = *1,482*)* were progressed for screening. A further (*n = *1,404) titles and abstracts were excluded, and *(n = *78) studies were sought for retrieval. Only (*n = *73) studies were assessed for full text eligibility and of these, (*n = *63) were excluded following full text screening according to our eligibility criteria (Supplementary Table 2). Ten studies (26–35) met the inclusion criteria and were included in the review (see Figure 1).

### 3.2. Study characteristics

The included studies were from four LMIC regions (Sub-Saharan Africa, South Asia, East Asia Pacific and Latin America and the Caribbean). Four studies were from Uganda (26–29), one study was from India (34), one study was from China (35), one study was from Mexico (33), one study was from Nigeria (30), one study was from Eswatini (32) and one study was from Ivory Coast (31) (Supplementary Figure 1). Eight of the ten studies were cross-sectional studies, one utilised mixed method approaches (a cross-sectional study combined with a qualitative study), and one was a qualitative study.

### 3.3. Collating, summarizing and reporting the results

All studies included adolescents and young adults with 4,293 who defined themselves as male. Two studies included younger adolescent men (30, 34), one study focussed on college age students (35), and the remaining studies focussed on those aged 18–24. The phenomenon of interest in all the studies was the association between SRH *(age at sexual debut, sexual coercion, previous sexual experience, number of sexual partners, condom use, transactional sex history, unmet sexual health counselling needs, gender assessment, sexual behaviour stigma, alcohol use in the context of sex, sexual initiation, HIV and healthcare, secondary sexual characteristics, sex hormone determination and same-sex sexuality)* and mental health (*anxiety, depression, post-traumatic stress disorder, suicidal ideation, emotional health)* (Supplementary Figure 2)*.* There were differences in the type of SRH and mental health outcomes included as well as in the method of measurements used to assess these outcomes. In addition, some studies reported on more than one of the SRH and mental health outcomes.

Four studies used the Hopkins Symptom Check List-25 (HSCL-25) developed by (36) and the Symptom Checklist-90 (SCL-90) developed by (37) to measure mental health outcomes *(anxiety and depression)* (26–29). One study used the Corah Dental Anxiety Scale to measure *dental anxiety* (30), whilst the others used the Patient Health Questionnaire (PHQ-9) to measure *depression* (32), the General Health Questionnaire (GHQ) to assess *mental health* (34), the Center for Epidemiologic Studies Depression scale (CES-D) to measure *depression* (31) and the Self-rated Depression Scale (SDS) to measure *depressive symptoms* (35). One study included outcomes on post-traumatic stress disorder (PTSD) and measured the outcome using a 7-item screening tool (31). Eight studies used a self-reported questionnaire to measure SRH and mental health outcomes (26–32, 34). One study conducted qualitative interviews with the participants (33) whilst another study incorporated focus group discussions as part of a mixed methods study (34). Additionally, one study used a physical examination (to assess secondary sexual characteristics) and a diagnostic test (to assess semen quality) as a method of measurement (35).

Characteristics of the included studies outlining the key findings are shown in Table 1.

**Table 1:** Characteristics of included studies.

First author, year & country	Study design	Sample size & setting	Outcome(s) of interest	Methods of measurement	Summary of key findings on SRH and mental health
Agardh (26), Uganda	Cross-sectional study	633 male students, (64.6% of study population) at a public university.	SRH outcomes (*age at sexual debut, experience of sexual coercion, previous sexual experience, number of sexual partner &, condom use*)	Questionnaire	High scores on mental health (denoting poor mental health) were associated with early sexual debut. Stronger association in males [OR 1.6, 95% CI (1.1–2.5)] when compared to females [OR 1.3, 95% CI (0.7–2.5)]. Mental health was an effect modifier between the experience of sexual coercion and multiple sexual partners.
			Mental health outcomes (*depression & anxiety*)	HSCL-25 and the SCL 90	
Agardh (27), Uganda	Cross-sectional study	633 male students, (64.6% of study population) at a public university.	SRH outcomes (*previous sexual experience, number of sexual partners & condom use*)	Questionnaire	High scores on mental health (denoting poor mental health) were associated with higher rates of having previously had sex. The association was stronger in males [OR 1.5, 95 CI (1.04–2.1)] when compared to females [OR 1.4, 95% CI (0.9–2.2)]. High scores on mental health (denoting poor mental health) were associated with an increased number of sexual partners with the association being stronger in males [OR 2.4, 95% CI (1.5–3.9)] when compared to females [OR 2.2, 95% CI (0.96–4.9)]. Results from the depression subscale indicated that high scores were associated with an increased number of sexual partners for both males [OR 2.4, 95% CI (1.5–3.8] and females [OR 3.0, 95% CI 1.3–7.1]. Association between elevated scores on anxiety and an increased number of sexual partners was stronger for males [OR 1.9, 95% CI (1.2–3.0)] when compared to females [OR 1.1, 95% CI (0.5–2.4)]. Association between inconsistent condom use and anxiety was stronger for males [OR 3.0, 95% CI (1.1–3.6)] when compared to females [OR 2.0, 95% CI (0.9–4.1)].
			Mental health outcomes (*depression & anxiety*)	HSCL-25 and the SCL 90	
Agardh (28), Uganda	Cross-sectional study	633 male students, (64.6% of study population) at a public university.	SRH Outcomes (*same-sex sexuality, number of sexual partners, transactional sex history, condom use & unmet sexual health counselling needs*)	Questionnaire	Poor mental was higher in participants who reported having sexual relations with someone of the same sex in 2005 at 75.3% [OR 3.1, 95% CI (1.8–5.3)] when compared to those who reported no sexual relations with someone of the same sex at 49.9%, and again in 2010 at 61.0% [OR 1.5, 95% CI (1.0–2.3)] when compared to those who reported no sexual relations with someone of the same sex at 51.0%.
			Mental health outcomes (*depression & anxiety*)	HSCL-25 and the SCL 90	
Folayan (30), Nigeria	Cross-sectional study	598 adolescent males, (56.6% of study population) in a semi-urban community.	SRH Outcomes (*history of sexual abuse, age of sexual debut, previously had sex, transactional sex history, number of sexual partners & condom use*)	Questionnaire	Probability of having high dental anxiety higher in those with a history of sexual abuse [OR_adjusted_ 1.81, 95% CI (1.10–2.98)] when compared to those with no history of sexual abuse. Results not stratified by gender and figures for latter group not specified.
			Mental health outcomes (*dental anxiety*)	CDAS	
Kyagaba (29), Uganda	Cross-sectional study	1,087 males (55.6% of study population) at a public university.	SRH outcomes (*age at first sexual encounter, number of sexual partners, condom use & experience of sexual coercion*)	Questionnaire	Unmet sexual health needs (a delay in seeking counselling for a sexual health problem) associated with high mental health scores (denoting poor mental health) for males and females [OR_crude_ 3.8, 95% CI (3.4–4.8); OR_adjusted_ 2.1, 95% CI (1.5–3.9)].
			Mental health outcomes (*feeling of loneliness, depression & anxiety*)	Question, HSCL-25 and the SCL 90	
Lyons (32), Eswatini	Cross-sectional study	532 adult males (100% of study population) in urban cities and towns.	SRH outcomes (*gender assessment, sexual behaviour stigma, number sexual partners & condom use*)	Questionnaire	Depression associated with high sexual behaviour stigma among participants with high stigma but not out [OR_adjusted_ 3.14, 95% CI (1.50–6.55)] when compared to those who had high stigma, and were out [OR_adjusted_ 2.42, 95% CI 1.51–3.87)]. Statistically significant difference *p* < 0.01.
			Mental health outcomes (*depression)*	PHQ-9	
Patel (34), India	Mixed methods study (cross-sectional study and qualitative study)	429 male students (53% of study population) at a secondary school.	SRH outcomes (*consensual sexual behaviours & experience of sexual abuse*)	Questionnaire	Male and female adolescents who experienced CSI have far worse mental health outcomes. Stronger association in girls [OR_adjusted_ 2.9, 95% CI (1.2–6.9)] when compared to boys [OR_adjusted_ 2.6, 95% CI (1.1–5.7)]. Suicidal ideation (within the past month) also higher among adolescents with a history of sexual abuse.
			Mental health outcomes (*suicidal ideation*)	GHQ	
Peltzer (31), Ivory Coast	Cross-sectional study	412 male students (50% of study population) at a public university.	SRH outcomes (sexual abuse, alcohol use in the context of sex, number of sexual partners & condom use)	Questionnaire	Depression associated with increased number of sexual partners in men [OR_crude _1.88, 95% CI (1.00–3.48)] when compared to women [OR_crude_ 1.55, 95% CI (0.81–2.93)].
			Mental health outcomes (post-traumatic stress disorder & depression)	7 item screening tool and CES-D	
Verduzco (33), Mexico	Qualitative study	15 adult males (100% of study population) in an urban area.	SRH themes (*sexual initiation, multiple sexual partners, HIV and healthcare & condom us*e)	Questions answered in interviews.	General anxiety associated with the first sexual encounter among MSM.
			Mental health themes (*emotional health*)		
Zou (35), China	Cross-sectional study	587 male students (100% of study population) at a public university.	SRH outcomes (*secondary sexual characteristics, semen quality & sex hormone determination*).	Physical examination and semen sample analysis.	Sperm concentration (×10^6^/ml) lower in depressed men [median 48.1, 5th–95th percentile (8.5–231.3), mean 66.9, SD (74.5)]. Total sperm count (×10^6^) also lower in depressed men [median 156.67, 5th-95th percentile (20.0- 962.9), mean 241.6, SD (299.7), *p* = 0.024]. No substantial differences found between depression and the levels of serum reproductive hormones among participants.
			Mental health outcome (*depression)*	SDS	
First author, year & country	Study design	Sample size & setting	Summary of key findings on other related outcomes		
Agardh (26), Uganda	Cross-sectional study	633 male students, (64.6% of study population) at a public university.	Having consumed alcohol at the last sexual encounter was associated with early sexual debut. Stronger association in males [OR 3.2, 95% CI (1.7–6.2)] when compared to females and [OR 1.8, 95% CI (0.7–4.8)]. Quadruple risk of increased of multiple sexual partners in males who consumed alcohol at the last sexual encounter [OR 7.2, 95% CI (3.5–15.0)] when compared to females [OR 2.4, 95% CI (0.8–7.0)].		
Agardh (27), Uganda	Cross-sectional study	633 male students, (64.6% of study population) at a public university.	Frequent heavy episodic drinking (FHED) (≥6 glasses) associated with multiple sexual partners in males [OR 3.5, 95% CI (1.3–9.5)] and females [OR 3.5, 95% CI (1.3–9.5)].		
Agardh (28), Uganda	Cross-sectional study	633 male students, (64.6% of study population) at a public university.	Frequent heavy episodic drinking (FHED) (≥6 glasses) higher among participants who reported having sexual relations with someone of the same sex in 2005 at 32.9% [OR 2.2, 95% CI (1.2–4.0)] when compared to those who reported having no sexual relations with someone of the same sex at 20.3%, and again in 2010 at 41.9% [OR 1.7, 95% CI (1.02–2.7)] when compared to those who reported having no sexual relations with someone of the same sex at 29.4%.		
Folayan (30), Nigeria	Cross-sectional study	598 adolescent males, (56.6% of study population) in a semi-urban community.	Probability of high dental anxiety higher in adolescents reporting alcohol intake (daily, <1 a week, weekly) OR_adjusted_ [1.74, 95% CI (1.19–2.56)].		
Peltzer (31), Ivory Coast	Cross-sectional study	412 male students (50% of study population) at a public university.	Heavy episodic drinking (HED) associated with multiple sexual partners in men (≥5 drinks) [OR 2.66, 95% CI (1.45–4.88)] when compared to women (≥4 drinks) [OR 1.55, 95% CI (0.81–2.93)].		

CDAS, corah dental anxiety scale; CES-D, center for epidemiologic studies depression; CI, confidence interval; CSI, coercive sexual intercourse; FHED, frequent heavy episodic drinking; GHQ, general health questionnaire; HED, heavy episodic drinking; HIV, human immunodeficiency virus; HSCL-25, hopkins symptom check list-25; ml, millilitre; MSM, men who have sex with men; OR, odds ratio; OR_adjusted_, adjusted odds ratio; OR_crude_, crude odds ratio; *p*, *p*-value; PHQ-9, patient health questionnaire; SCL-90, symptom checklist-90; SD, standard deviation; SDS, self-rated depression scale.

## 4. Discussion

### 4.1. Key findings

The scoping review aimed to investigate what is known about the association between SRH and mental health of young men aged 10–24 in LMICs. Findings suggest there is a reciprocal interaction between mental health and SRH. In particular, sperm concentration and total sperm count was lower in depressed men (35). Poor mental health was associated with early sexual debut (26), higher rates of having previously had sex and an increased number of sexual partners (27). Depression and anxiety specifically, were also associated with an increased number of sexual partners (27, 31), and anxiety was also associated with inconsistent condom use (27).

The interaction between mental health and sexual abuse was also highlighted. Dental anxiety was associated with a history of sexual abuse (30) and adolescent males who experienced coercive sexual intercourse experienced poor mental health. Furthermore, suicidal ideation increased among those male adolescents with a history of sexual abuse (34). Findings further suggest that a delay in seeking counselling for a sexual health problem was associated with poor mental health (29). Our results also indicate that alcohol use at the last sexual encounter was associated with multiple sexual partners (26, 27, 31).

### 4.2. Comparison to current literature

The association between semen quality and poor mental health was consistent with findings from (38, 39). These studies found that sperm concentration and sperm count were lower in males who were experiencing depression and anxiety. Although the findings from the review suggest that there were no differences between depression and the levels of serum reproductive hormones, this was contradicted by the (38) study which found that testosterone levels were lower in men who experience anxiety and depression.

The relationship between poor mental health, early sexual debut and the experience of having previously had sex was consistent with findings from previous studies (40, 41). Kastbom (41) found that adolescent boys who reported symptoms of depression and anxiety had a higher likelihood of experiencing early sexual debut [OR 2.63, 95% CI (1.64–4.29)] when compared to adolescent boys who reported no symptoms of depression and anxiety. Similarly, Meier (40) found that adolescents who had early sexual debut and split up with their partner had a higher likelihood of experiencing depressive symptoms.

The correlation between poor mental health and increased number of sexual partners among males was consistent with findings from (42). The study found that adolescents with psychiatric problems particularly those who were in the “mania” category had a higher probability of reporting two or more sexual partners [OR 3.2, 95% CI (1.1–9.5)]. Although the results in this study were not stratified by gender.

The relationship between alcohol and sexual behaviours has been documented before and that men are more likely to self-medicate using alcohol or other substances when experiencing mental health problems (8). Furthermore (43), found that participants who abused alcohol were more likely to report higher levels of depressive or additional psychiatric symptoms. Previous studies have documented the link between SRH and alcohol (44, 45). Further research would be required to establish the causal relationship between alcohol abuse, SRH and poor mental health.

SRH legislation and policies also impact on mental health. For instance, there are 67 United Nations member states that criminalise private consensual same-sex acts among adults (31 in Africa, 9 in Latin America and the Caribbean, 22 in Asia and 6 in Oceania) (46). The concern is that the SRH needs of MSM remain unmet which will potentially lead poor mental health outcomes. According to (47), the health needs of men who have sex with men (MSM) are often disregarded in regions where homosexuality is forbidden. In addition, the criminalisation of same-sex acts has been found to be associated with higher levels of perceived sexual stigma (48). Our findings suggest that poor mental health was higher in MSM and that depression was significantly higher in males who experienced sexual behaviour stigma (28, 32). In addition, the first sexual encounter among MSM was linked to anxiety (33). This could partially explain why we found that frequent heavy episodic drinking was higher among MSM (28).

### 4.3. Strengths and limitations

The review was conducted with rigour and was reported using the PRISMA-ScR checklist. Developing comprehensive search strategies and using two additional reviewers to pilot the rigorous eligibility criteria enhanced the credibility of the results. To our knowledge, this is the first review to map out the association between SRH and mental health among young men in LMICs. Considering that the included studies are from four LMIC regions, this enabled a study of a relatively heterogenous population.

Although the results point to the fact that poor mental health is associated with poor SRH outcomes and vice versa, it was difficult to separate temporality. This was due to the study designs of the included studies. Causality cannot be inferred because of the absence of temporal elements particularly in cross-sectional study designs (49). Therefore, we cannot ascertain the direction of the association between SRH and mental health which is limitation of this study. Additionally, the exclusion of grey literature and studies not in English implies that some studies meeting the eligibility criteria may have been missed. Finally, the different mental health measurement tools made it difficult to make comparisons between studies and could have led to an overestimation or underestimation of the reported mental health symptoms. Thus, these findings need to be interpreted with caution.

### 4.4. Implications for research and practice

The findings confirm a paucity of studies investigating the link between SRH and mental health in men. This hinders the understanding of the dynamic interaction between these important issues. Consequently, there is a need for well-designed studies that will map the temporal association between SRH and mental health among young men.

Most young adults in LMICs have limited or no access to SRH education and services (50). In addition, evidence suggests that adolescent males are less aware of mental health services (51). Lack of access to information about SRH and mental health may potentially act as a barrier and hamper adolescent males’ ability to acknowledge their need to access SRH and mental health services. Therefore, there is a need for more research on intervention strategies that can be employed to mitigate this challenge.

Although there was heterogeneity in the location of the studies, 70% occurred in an education setting with students. These study populations may not be a true representation of the population of young men in LMICs considering that those not enrolled in educational institutions will be excluded. Therefore, there is a compelling need to conduct further research in different contexts and with diverse populations. Future studies should ensure that data on SRH and mental health is collected at national levels across LMICs. This research could be key in informing national SRH and mental health policy.

## 5. Conclusion

The findings of our review suggest that poor mental health is associated with poor SRH outcomes and that poor SRH outcomes are associated with poor mental health among young men (10–24) living in LMICs. Further research is needed to establish any causal relationship between the different SRH and mental health outcomes and should include a wide range of participants.
