# Supplementary material for: Sexual, reproductive and mental health among young men (10–24) in low-and-middle income countries: a scoping review
Source: Front Reprod Health. 2023 Dec 4;5:1119407. doi: 10.3389/frph.2023.1119407 (PMC10725937; doi:10.3389/frph.2023.1119407)
Supplement: Supplementary file 1 [file Datasheet1.docx]

Supplementary Material

Sexual, reproductive and mental health among young men (10-24) in low-and-middle income countries: A scoping review

Siphiwe Mhlongo^1^, Amanda J. Mason-Jones^1*^ Keith Ford^1^

^1^ Department of Health Sciences, University of York, York, England.

*** Correspondence:**Corresponding Author
amanda.mason-jones@york.ac.uk

Content

[Supplementary Table 1. Preferred Reporting Items for Systematic reviews and Meta-Analyses extension for Scoping Reviews (PRISMA-ScR) Checklist. 3](#_Toc147494132)

[Supplementary Table 2. Eligibility Criteria. 5](#_Toc147494133)

[Supplementary Figure 1. Geographical location and the number of studies (n) from each country. 6](#_Toc120641007)

Supplementary Figure 2. Pie chart showing different SRH and mental health outcomes......................7

Supplementary Table 1. Preferred Reporting Items for Systematic reviews and Meta-Analyses extension for Scoping Reviews (PRISMA-ScR) Checklist.

| **SECTION** | **ITEM** | **PRISMA-ScR CHECKLIST ITEM** | **REPORTED**  **ON PAGE #** |
| --- | --- | --- | --- |
| **TITLE** | | | |
| Title | 1 | Identify the report as a scoping review. |  |
| **ABSTRACT** | | | |
| Structured summary | 2 | Provide a structured summary that includes (as  applicable): background, objectives, eligibility criteria,  sources of evidence, charting methods, results, and  conclusions that relate to the review questions and  objectives. |  |
| **INTRODUCTION** | | | |
| Rationale | 3 | Describe the rationale for the review in the context of  what is already known. Explain why the review  questions/objectives lend themselves to a scoping  review approach. |  |
| Objectives | 4 | Provide an explicit statement of the questions and  objectives being addressed with reference to their key  elements (e.g., population or participants, concepts, and  context) or other relevant key elements used to  conceptualize the review questions and/or objectives. |  |
| **METHODS** | | | |
| Protocol and registration | 5 | Indicate whether a review protocol exists; state if and  where it can be accessed (e.g., a Web address); and if  available, provide registration information, including the  registration number. | N/A |
| Eligibility criteria | 6 | Specify characteristics of the sources of evidence used  as eligibility criteria (e.g., years considered, language,  and publication status), and provide a rationale. |  |
| Information sources* | 7 | Describe all information sources in the search (e.g.,  databases with dates of coverage and contact with  authors to identify additional sources), as well as the  date the most recent search was executed. |  |
| Search | 8 | Present the full electronic search strategy for at least 1  database, including any limits used, such that it could be repeated. |  |
| Selection of  sources of evidence† | 9 | State the process for selecting sources of evidence (i.e.,  screening and eligibility) included in the scoping review. |  |
| Data charting process‡ | 10 | Describe the methods of charting data from the included  sources of evidence (e.g., calibrated forms or forms that  have been tested by the team before their use, and  whether data charting was done independently or in  duplicate) and any processes for obtaining and  confirming data from investigators. |  |
| Data items | 11 | List and define all variables for which data were sought  and any assumptions and simplifications made. |  |
| Critical appraisal of  individual sources  of evidence§ | 12 | If done, provide a rationale for conducting a critical  appraisal of included sources of evidence; describe the  methods used and how this information was used in any  data synthesis (if appropriate). | N/A |
| Synthesis of results | 13 | Describe the methods of handling and summarizing the  data that were charted. |  |
| **SECTION** | **ITEM** | **PRISMA-ScR CHECKLIST ITEM** | **REPORTED**  **ON PAGE #** |
| **RESULTS** | | | |
| Selection of  sources of evidence | 14 | Give numbers of sources of evidence screened,  assessed for eligibility, and included in the review, with  reasons for exclusions at each stage, ideally using a flow diagram. |  |
| Characteristics of  sources of evidence | 15 | For each source of evidence, present characteristics for  which data were charted and provide the citations. |  |
| Critical appraisal  within sources of evidence | 16 | If done, present data on critical appraisal of included  sources of evidence (see item 12). | N/A |
| Results of  individual sources  of evidence | 17 | For each included source of evidence, present the  relevant data that were charted that relate to the review  questions and objectives. |  |
| Synthesis of results | 18 | Summarize and/or present the charting results as they  relate to the review questions and objectives. |  |
| **DISCUSSION** | | | |
| Summary of evidence | 19 | Summarize the main results (including an overview of  concepts, themes, and types of evidence available), link  to the review questions and objectives, and consider the  relevance to key groups. |  |
| Limitations | 20 | Discuss the limitations of the scoping review process. |  |
| Conclusions | 21 | Provide a general interpretation of the results with  respect to the review questions and objectives, as well  as potential implications and/or next steps. |  |
| **FUNDING** | | | |
| Funding | 22 | Describe sources of funding for the included sources of  evidence, as well as sources of funding for the scoping  review. Describe the role of the funders of the scoping review. |  |

Supplementary Table 2. Eligibility Criteria.

| - **Domain** | - **Description** |
| --- | --- |
| - **Setting** | - All studies must be from low-and-middle-income countries (LMICs) as per World Bank 2023 fiscal year classification. |
| - **Perspective** | - ≥50% of participants must be male and aged 10 – 24. - All studies must have participants that are human. |
| - **Phenomenon of Interest** | - All studies must include data on sexual and reproductive health and mental health. |
| - **Evaluation** | - All study designs apart from commentaries, letters, case studies and conference abstracts. |


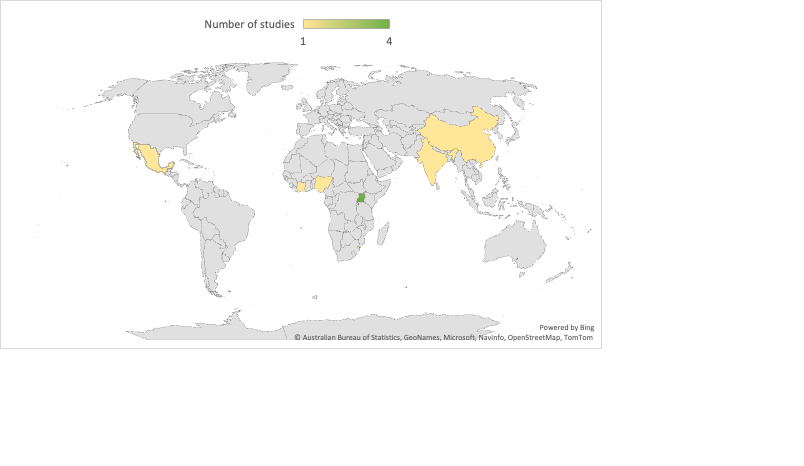


Mexico (n=1)

Ivory Coast (n=1)

Nigeria (n=1)

India (n=1)

China (n=1)

Eswatini (n=1)

Uganda (n=4)

Supplementary Figure 1. Geographical location and the number of studies (n) from each country.

Supplementary Figure 2. Pie chart showing different SRH and mental health outcomes.
